# Robert Arthur, M. D., D. D. S.

**Published:** 1880-09

**Authors:** W. W. H. Thackston


### ARTICLE III.

### *Robert Arthur, M. D., D. D. S.*

BY W. W. H. THACKSTON, OF VIRGINIA.

Private advices, and the professional Journals, have brought to us the announcement of the death of Dr. Robert Arthur, of the city of Baltimore.

This sad event occurred July 23rd of the present year, in the fortieth year of a professional career, in which Dr. Arthur stood in the fore front of that wonderful progress, which has marked the four past decades of dental history. Forty years ago, the Baltimore College of Dental Surgery (then the only institution of its kind in the world,) presented as its first offering to science, Robert Arthur; and had that college, whose subsequent labors and influence have remodeled, and reconstructed, and elevated and advanced dental science and dental practice throughout the world, done nothing more than produce *one* such representative of its teachings, that enterprise would not have been founded in vain, but would have deserved, and commanded the admiration and applause of men, and fully compensated all the efforts and labors for its establishment.

Immediately after graduating, Dr. Arthur commenced the practice of his profession ; and very soon took high rank as a skillful operator, a close observer, and faithful student. His research and studentship did not end with his graduation, but only terminated with a life of indefatigable industry and enlarged usefulness.

Such was Dr. Arthur's standing and reputation that even in this early period of his professional career —while just in the dame of his manhood,—he commanded the recognition and engaged the confidence and esteem of the leading members of a profession than struggling for improvement for position, and for legal existence. The success of the school in Baltimore, having demonstrated the value and importance of the system upon which this former institution was founded; it was determined to establish a similar school in the city of Philadelphia, and it was no surprise to the dentists of that day, that Dr. Arthur was promptly called to fill one of the most important professorships in the "*Pennsylvania College of Dental Surgery*;" nor was it a surprise to any one acquainted with his talents and acquirements, that the duties of his responsible position were so discharged, as to reflect the highest honor upon him as a teacher, to enhance the reputation of the school, and to confer the largest benefits and advantages upon the classes to whom he lectured.

Professor Arthur's position as an instructor and educator, extended his acquaintance with men of science, brought him into association with savans and scholars, increased his already high reputation, and enlarged the sphere of his professional practice. After several years of successful and brilliant service as a leading member of the Faculty of Instruction in the Pennsylvania College, Dr. Arthur found himself antagonized by some of his colleagues (as well as we now remember) in respect to questions of an ethical character, in the conduct and management of the school and the remonstrance of the "Board of Visitors," and to the profound regret of the students and patrons of the college, he resigned his Professorship, and permenantly terminated his connection with the educational institutions of his profession, except as to the various associations and conventions of which he remained an honored and highly distinguished member, to the end of his life.

Dr. Arthur's talents and abilities found ready recognition and acknowledgement abroad; and the Odontological and other societies of Europe, honored themselves by confering upon him the highest distinctions within their gift. His views were eagerly sought and discussed, and his improvements and advances largely accepted and adopted.

From many influential sources, Dr. Arthur had been urged to relinquish his connection with the Pennsylvania College, before he finally determined to submit his resignation as a professor. Finally, he yielded to the importunities of his Baltimore friends in so far as to return to the "Monumental City" and there to spend the remainder of his busy life, dispensing the benefits of his skill and learning to those who sought his services. And we do not think it extra urgent to say, that few, if any, ever enjoyed a higher or more appreciative class of patrons in the history of American Dentistry.

But amid all the stretch and strain upon his strength and energies and as a popular and approved operator, Dr. Arthur never relaxed his efforts from the growth and development of his loved profession. He continued *to study, to think, and to invent.* The current literature of the profession has for nearly forty years, enriched by contributions from his facile pen, and many valuable additions have been made to our stock of knowledge, to the instruments, the material, and the resources of operative dentistry by his fertile and teeming brain.

In this hurried sketch, we cannot recall attention to all the many valuable papers attributed to dental science by our lamented friend, but will content ourself with the mention of a few, which suggest themselves as we write. The exhaustive report upon the "Dental Literature of the World" made before the old "American Society of Dental Surgery," in the year A. D. 1850 or 1851, was a paper of great research and accuracy, and of inestimable value to the student of science. This report was written in a style that stamped Dr. Arthur as a man of broad professional culture, and of classic skill and taste in the realm of letters. Subsequently the publication of his monograph upon the "adhesive properties of annealed gold foil," giving to dental practice, essentially a *new material*, or rather an old material so modified in its character, as to largely supersede all other forms of gold, and revolutionize the grand operation of "tooth-filing" the world over, added to the high reputation its author had already achieved. Many other articles and essays having the stamp of thought, of research, and of genius, may be found illuminating the pages of our current journalism, but we have neither time or space to comment upon these in this article.

Perhaps the most noted and important of all Dr. Arthur's contributions to the profession he so highly honored and adorned, was his work on the "Treatment and Preservation of the Teeth."

The form in which this book was given to the profession, provoked at the time of its appearance, the sharp criticism of some of the "journals," both at home and abroad. The *popular* style adopted by the author, was regarded as objectional, and as a departure from strictly professional usage, and the doctrine taught and maintained, did not receive the sanction or approval of many of the leading practitioners of the day. But, however well founded some of these objections and criticisms may have been, it nevertheless cannot be denied, that the work was a great addition to the literature and operative resources of dental science, that it evinced close observation, patient investigation, a high order of talent, and a most refined and cultivated scholarship.

It may now be safely asserted, that the prophylaxis and treatment of decay of the teeth as taught by Dr. Arthur, has been largely adopted by many of the most distinguished and reputable dentists of the world, and that the results of such treatment and practice, have abundantly demonstrated its value and importance in the salvation of human teeth. In making this assertion, I mean the *true and exact teachings of the work*, *not* the crude conceptions and total misapprehensions of Dr. Arthur's theory and practice that we have seen published in some of the Journals and Reviews, *not* the muddy and opaque ideas that have occasionally found flippant expression in our *societies* and *conventions*, but a clear understanding of the author's meaning, and a faithful application of the principles, the methods, and the operations prescribed.

No man living or dead, has contributed more to perfect the operation of "tooth-filling," than Dr. Arthur; no one had a higher appreciation of the value and effectiveness of that particular means of "tooth-saving," no one more unhesitatingly performed the operation whenever and wherever indicated, and few if any, performed the work with more skill and judgment, whether the material employed was soft gold or crystal gold, or hard adhesive gold foil. As far as the writer has had opportunity of examining Dr. Arthur's filling, he found them masterpieces of faithful finished workmanship. It was a total mistake that he disapproved the operation of "filling," or in any-way discouraged its employment. But Dr. Arthur believed and taught that with the best and highest efforts of human skill, and with the greatest attainable perfection in material, in instruments, and in operative ability, that a tooth filled upon its *proximate surfaces* was not as effectually preserved, or with equal value with one, in which decay had been anticipated and prevented, or having occurred, had by a timely operation of a different character been swept away, leaving sound, healthy, highly polished and self cleaning surfaces, with comparatively little mutilation, and scarcely appreciable impairment of the natural symmetry of the organ.

We suppose no one will deny to Dr. Arthur the credit and honor of discovering the peculiar properties of what is now known as "Annealed" or "Adhesive" gold foil which made possible and practicable, the filling, and building up and contouring, and preserving for indefinite usefulness, all decayed and broken down teeth capable of restoration by this operation. He so taught in his last book, and so practiced in his daily ministrations to the needs of his patients. Upon the grinding, the buccal, the lingual, and labial surfaces of the teeth-fillings of gold, glitter and sparkle all along his professional pathway, and upon the proximate surfaces, where the progress of decay made a filling necessary, there it will be found, in all the teeth that came under his eye and hand.

But however dentists may differ as to some of Dr. Arthur's advanced ideas of Theory and Practice, all will unite in the acknowledgement of his eminent ability, and unflagging industry ; all will acknowledge the debt of gratitude his labors and achievements have imposed, and all will embalm his memory, as one of the high and honored of our calling, as a good and a great man who has fallen at his post. It is no disparagement of our illustrious dead, or of our distinguished and honored living to say that Robert Arthur was the equal, the peer of the most exalted of them all ; and to-day, American Dentistry stands bereft of one of its brightest exemplars and grandest representatives. Alas! alas!

In the private and social relations of life, Dr. Arthur was no less a man of mark than in the public and professional positions which he so highly honored and adorned. With a great and warm heart, and a massive intellect strengthened, cultivated, and refined by life long study of the models of classic and modern genius, the languages and literature of the past and the present, he was a most instructive and charming companion, and with those who engaged his confidence and esteem, a most disinterested and devoted friend. Unostentatious, modest, and diffident almost to a fault, he made no parade of his extraordinary abilities, or brilliant acchievements, and it was often a source of painful and almost crushing disappointment that he found his highest and grandest efforts in behalf of his profession so often misapprehended and misinterpreted, by the very class of men, that he had labored and struggled to benefit and improve. But in the silence and the seclusion in which all sensitive natures take refuge, he worked on, trusting for his recompense and reward, to the consciousness of his integrity, and in the firm faith, that ultimately his work would be understood and appreciated ; his fellow men benefited and advantaged, and that posterity, would accord to him the justice, and honor, often withheld by his contemporaries. And so it will be.

The last few years have witnessed the fall of many of our high and honored men ; death has shot his arrows at our most shining marks, but it may be safely said, that no more distinguished representative of our profession, no more elevated or exemplary character, no truer or brighter exemplar of all that dignifies and ennobles humanity has been cut down, than Robert Arthur, of Baltimore. Peace to his ashes, and forever honored and cherished be his memory.

